# KGen: a knowledge graph generator from biomedical scientific literature

**DOI:** 10.1186/s12911-020-01341-5

**Published:** 2020-12-14

**Authors:** Anderson Rossanez, Julio Cesar dos Reis, Ricardo da Silva Torres, Hélène de Ribaupierre

**Affiliations:** 1 Institute of Computing, University of Campinas, Campinas, SP Brazil; 2 Department of ICT and Natural Sciences, Faculty of Information Technology and Electrical Engineering, NTNU – Norwegian University of Science and Technology, Ålesund, Norway; 3 School of Computer Science and Informatics, Cardiff University, Cardiff, UK

**Keywords:** Knowledge Graphs, RDF Triples, Ontologies, Information Extraction

## Abstract

**Background:**

Knowledge is often produced from data generated in scientific investigations. An ever-growing number of scientific studies in several domains result into a massive amount of data, from which obtaining new knowledge requires computational help. For example, Alzheimer’s Disease, a life-threatening degenerative disease that is not yet curable. As the scientific community strives to better understand it and find a cure, great amounts of data have been generated, and new knowledge can be produced. A proper representation of such knowledge brings great benefits to researchers, to the scientific community, and consequently, to society.

**Methods:**

In this article, we study and evaluate a semi-automatic method that generates knowledge graphs (KGs) from biomedical texts in the scientific literature. Our solution explores natural language processing techniques with the aim of extracting and representing scientific literature knowledge encoded in KGs. Our method links entities and relations represented in KGs to concepts from existing biomedical ontologies available on the Web. We demonstrate the effectiveness of our method by generating KGs from unstructured texts obtained from a set of abstracts taken from scientific papers on the Alzheimer’s Disease. We involve physicians to compare our extracted triples from their manual extraction via their analysis of the abstracts. The evaluation further concerned a qualitative analysis by the physicians of the generated KGs with our software tool.

**Results:**

The experimental results indicate the quality of the generated KGs. The proposed method extracts a great amount of triples, showing the effectiveness of our rule-based method employed in the identification of relations in texts. In addition, ontology links are successfully obtained, which demonstrates the effectiveness of the ontology linking method proposed in this investigation.

**Conclusions:**

We demonstrate that our proposal is effective on building ontology-linked KGs representing the knowledge obtained from biomedical scientific texts. Such representation can add value to the research in various domains, enabling researchers to compare the occurrence of concepts from different studies. The KGs generated may pave the way to potential proposal of new theories based on data analysis to advance the state of the art in their research domains.

## Background

Knowledge evolves over time, often fostered by new findings or by changes in adopted reasoning methodologies. Even by chance, new facts or evidences may become available, leading to new understandings about complex phenomena. This is particularly true for the medical domain, where scientists continuously work on finding new methods for diagnosis, treatment, and possibly, cure.

Regardless of the medical subarea, facts and findings about diseases have been documented extensively, opening new opportunities for knowledge acquisition and expansion. Examples include research outcomes written and published, for instance, in theses, dissertations, articles, reports, among other textual formats utilized in the scientific literature.

Scientific investigations generate a massive amount of data, from which new knowledge may be produced. This formidable increase of the amount of data available makes it now almost impossible for scientists to properly understand and extract new knowledge without help. We claim that the use of effective computational methods for creating knowledge representations is a suitable alternative to support scientific investigations in the medical domain. As an example, such representations could provide a way to correlate a concept from a specific work with other concepts from the same study, as well as with concepts from similar investigations. By observing such relations, researchers might be able to formulate new hypotheses or draw new conclusions, advancing therefore the state of the art in a research domain.

The building of such representations requires determining the facts that may be either explicit or even implicit in diverse portions of a scientific text. Facts can be extracted from sections, paragraphs, sentences, or even in parts of sentences. For instance, consider the sentence *Alzheimer’s Disease causes dementia*. One may observe from the sentence, that: (1) There is a disease named *Alzheimer’s Disease*; (2) This disease causes a condition named *dementia*; (3) *Alzheimer’s Disease* and *dementia* are entities; (4) There is a relation between such entities denoted by the verb *cause*. In this sense, knowledge may be represented by a set of facts considering a set of relations among entities. Considering the above observations, we came up with the following research question: *Is it possible to represent knowledge obtained from scientific texts, identifying biomedical entities associated with well-known concepts in the biomedical domain, and determine how such entities relate to each other?*

In this research, we propose the use of Knowledge Graphs (KGs) to represent knowledge extracted from scientific texts in natural language in the biomedical domain. KGs define the interrelations of entities in facts [1], modeling knowledge using the *Resource Description Framework* (RDF) [2] representation, relying on Linked Data principles [3]. Such principles play a central role in the in standardization and dissemination of the knowledge for several purposes. The key aspect is that RDF datasets must define links to external resources. Linked data technologies have become increasingly relevant for semantic interoperability and knowledge discovery in life sciences [4]. RDF datasets in life sciences, such as Bio2RDF [5], MeSH [6], and AGROVOC [7] are part of the Linked Open Data (LOD) cloud, following the Linked Data principles. Bio2RDF refers to one of the largest networks providing linked data in life sciences. It has been used, for instance, as a knowledge retrieval tool that supplies information about the Human Immunodeficiency Virus (HIV) [8]. The representation of life sciences’ knowledge in a linked data perspective has also derived several research topics and advancements. Some examples include the usage of KGs to integrate multiple types of life sciences’ data through queries in disease networks [9], creation of an Ebola centered Knowledge Base [10], challenges on using commercial data with linked data in pharmacological context for the discovery of new drugs [11], and the development of a linked dataset to aid doctors in choosing the best clinical treatment for patients [12]. In this sense, the computational representation and description of disease information by means of KGs might add great value to the analysis and understanding of diseases [13].

The generation of KGs from unstructured text through a completely automated procedure is still an open research problem. Considering biomedical scientific texts as input imposes further challenges to this problem. In such type of texts, we may find, among others, long and complex sentences containing implicit relations, abbreviations, and co-references to entities (through prepositions). The recognition of entities may require specific previous knowledge in the domain, turning it difficult for computational tools and techniques to perform such task automatically.

In this paper, we define and develop KGen, a KG generator from natural language texts from biomedical scientific literature using Natural Language Processing (NLP) techniques. We propose a semi-automatic method, in which an human (with specific domain expertise) may interfere with the process when required, ensuring the generation of suitable graphs.

In our method, KGs are generated by first identifying sentences from within a chunk of unstructured text, resolving abbreviations, co-references, and later simplifying sentences to obtain a set of small and cohesive sentences. For each sentence in the set, our solution detects and extracts information in form of RDF triples, constituted of a subject, a predicate, and an object. Such constituents are linked to classes, properties, and attributes from a biomedical ontologies. Finally, we build a graph representation by combining the set of triples with the set of obtained links.

This work extends and advances the investigation started by Rossanez and Dos Reis [14] in the following aspects: We improve the information extraction from textual sentences, in a way that not only the main, but also secondary information is extracted from sentences, resulting in a greater amount of RDF triples. The recognition of biomedical entities was improved. For this purpose, we link such entities to the Unified Medical Language System (UMLS) [15]. We refined the technique for Ontology linking using the obtained UMLS links as an intermediate step to acquire the final links for ontologies available in the NCBO bioportal [16]. Such novel aspects are incorporated into the *KGen* software tool, available at https://github.com/rossanez/kgen (As of Jan. 2020). In addition, we report further experimental results to assess the quality of the KGs generated from our proposal.

Performed validation considers the scenario of handling information about degenerative diseases. Degenerative diseases are the result of a continuous debilitating process in cells, that ends up affecting tissues and organs, becoming worse over time [17]. They may interfere with balance, movement, breath, and even the heart function [18] in an individual. One example of such degenerative diseases is the Alzheimer’s Disease (AD). AD is one of the leading causes of death throughout the world, especially among individuals aged 65 and older [19]. It is estimated to begin manifesting at least 20 years before the initial symptoms are noticeable [20]. Such symptoms examples are memory loss and language difficulties, which increase over time, up to a point where the individual is said to present dementia caused by AD [21]. The disease is not yet curable.

We conduct an evaluation involving physicians, keen to scientific texts on the biomedical domain related to AD. We handed them abstracts from scientific papers on AD, and asked them to manually extract triples from such texts. We then compared their triples to the ones extracted using our proposed method. Physicians were involved in carrying out a qualitative assessment of the generated KGs from our solution. In addition, we compared the ontology linking results from our previous work against the specific linking method that we present in the current work. The results show that, through our method, a significant amount of triples is extracted in comparison with the manually extracted ones. The proposed linking method in this work is capable of finding more links than the method described in our previous work. Experimental results indicate the quality of the KGs generated.

In summary, this work presents the following contributions:

- A new semi-automatic method that generates KGs linked to biomedical ontologies, from unstructured biomedical scientific texts;
- A rule-based technique that extracts the main relation from a sentence, based on the main verb and its arguments. The technique derives secondary relations from the same sentence, seeking compositions, adjectives, and modifying relations. This results in a more detailed KG representation;
- A technique that finds links in biomedical ontologies through SPARQL queries [22] with the use of UMLS mappings;
- A software tool developed and available to generate such linked KGs, by fully implementing the proposed method and techniques.

Studies dealing with KG building from unstructured text generally subdivide such main task into smaller sub tasks. At first, identifying or extracting valuable information from text, and then modeling such information into RDF triples, constituted of a subject, a predicate, and an object. Another task refers to link concepts or entities represented in triples into knowledge bases for the generation of linked KGs. The following subsections describe and discuss existing techniques and studies addressing such issues.

### Information extraction

The information extraction from text is an important and challenging task. Several NLP tools [23] and techniques are usually employed in this process, e.g., Part of Speech (PoS) taggers, constituency and dependency parsers, and Named Entity Recognition (NER).

A common approach on information extraction consists in identifying entities and verbs in a sentence. These would be the subject, object, and predicate candidates of an RDF triple. Verbs are identified by PoS tagging a sentence. Entities, on the other hand, may be identified using NER. This technique identifies entities within a sentence, and it is usually implemented using a classifier [24] that assigns the entities into categories (e.g., person, organization, etc.). Such classifier requires a trained model to identify the named entities. This way, it is domain-dependent. Using it in biomedical text requires a model trained in that domain, for which it is necessary to have a considerable amount of manually annotated text.

A possible strategy for extracting information relies on the use of open information extraction systems. Such systems rely purely on the identification of lexical and syntactical patterns in text sentences. Some examples of open information extraction systems are *ReVerb* [25], *OLLIE* [26], *Stanford OpenIE* [27], and *ClausIE* [28]. One advantage of such systems is that, since they rely on sentence patterns, they are not domain-dependent. However, on the other hand, they may fail on identifying important information, or even identifying erroneous relations in case of complex sentences (e.g., containing conditionals), or in passive voice sentences.

Semantic Role Labeling (SRL) [29] is a technique widely used in information extraction. It identifies the semantic roles of a verb in a given sentence (e.g., Agent, Patient, and Theme). For instance, in the sentence *Rosie eats vegetables*, the verb *eat* has two arguments, *Rosie* and *vegetables*. Such arguments assume the two semantic roles of the verb *eat*: an Agent (*Rosie*), and a Patient (*vegetables*). The roles are determined by consulting manually built lists of verb role sets for the English language, such as *VerbNet* [30], *PropBank* [31], and *FrameNet* [32].

Other approaches rely on machine learning techniques to identify entities and their relations within NL texts. Collobert et al. [33] employed neural network models in the *SENNA* system, which integrates PoS, NER, and SRL tasks. In most of the machine learning (or deep learning) approaches used for relation extraction, the identification is handled as a classification problem (i.e., determining the probabilities of a given relation from a sentence for a set of predefined relation types). Several neural network types have been used in these investigations, such as Recurrent Neural Networks (RNN) [34], and Convolutional Neural Networks (CNN) [35].

Li et al. [36] evaluated different neural models for biomedical entities and relation recognition. Such models present a great effectiveness in performing their job, but they require several amount of textual data to be properly trained in a particular domain. Furthermore, there are several hyper-parameters (e.g., number of hidden layers, and number of neurons in each layer) that need to be tuned, aiming at producing optimal outputs. For that, a significant amount of time and hardware power may also be required.

### Knowledge bases and ontology linking

A relevant task refers to linking extracted RDF triples to entities and concepts represented in ontologies to turn the semantic encoding of RDF resources formal and explicit. There are several well-known bases that are fit for such task. For instance, *DBpedia* [37], which contains structured information extracted from Wikipedia. It provides a SPARQL endpoint, as well as REST APIs, allowing queries to its structured dataset. Similar bases that may be used for the same purposes are, for instance, *Babelfy* [38], and *TagMe* [39].

Considering the biomedical domain, the National Center for Biomedical Ontology (NCBO) [16] provides an endpoint to access multiple biomedical ontologies, e.g., National Cancer Institute Thesaurus (NCIT), or Alzheimer’s Disease Ontology (ADO). It provides an annotator for natural language sentences, helping to identify mappings from concepts and entities to existing ontologies; as well as SPARQL and REST API endpoints. They have employed a recommendation service that suggests which ontology might contain the higher amount of matches for a given text. Their mapping service supports the alignment of concepts and properties between ontologies. Furthermore, each concept from ontologies contain a Concept Unique Identifier (CUI) field, mapped to the Unified Medical Language System (UMLS) [15], a semantic network that connects a concise collection of controlled vocabularies in the biomedical domain.

### KG building studies

We analyze literature studies that aim at building KGs and linking them to knowledge bases. Martinez-Rodrigues et al. [40] combined open information extraction systems and SRL to extract triples by means of a technique that considers noun phrases in the identification of entities. The identified entities are mapped to multiple knowledge bases, such as *DBpedia* [37], *Babelfy* [38], and *TagMe* [39]. Exner and Nugues [25] interconnected the extracted information to *DBpedia* [37], using a rule-based approach. In such investigations, if there is not an exact match for any of the triple’s constituents in the knowledge bases, such are left unmapped.

Exner and Nugues [41] considered NER combined with SRL techniques to assign the named entities as either subject or object, using the identified verb roles. This is very helpful, for instance, in passive voice sentences, where the subject and the object may have their orders changed in the RDF triples if using open information extraction systems, for instance.

Similarly, T2KG tool [42] explored a hybrid of a rule-based approach and a vector-based similarity metric to identify similar mappings to *DBpedia* [37] in case of a missing exact match.

FRED tool [43] generates its own ontology from a text, mapping existing entities and concepts to other existing knowledge bases, such as *DBpedia* [37]. It uses SRL combined with frame semantics [44] to extract relations from the text and build a graph.

PIKES [45] extracts information using SRL combined with a rule-based strategy to generate RDF triples, adding links to concepts found in bases such as *DBpedia* [37].

Other software tools have been proposed for the purpose of KG building. For instance, IBM provides a tool for the information extraction from plain text to ultimately build a KG integrating input documents [46]. The tool integrates a set of their services (e.g., Watson [47] and Cloud [48]).

### KGs in the biomedical domain

Knowledge graphs have been extensively exploited in the biomedical domain. Existing initiatives range from the proposal of new approaches to support knowledge acquisition through user interaction [12, 49] to the use of learning mechanisms to support the creation of effective classification and search tasks [50, 51].

The work of Ruan et al. [12], for example, introduced QAnalysis, a question-answering tool to support the analysis of large volumes of medical data. Users may define information needs in natural language, and the system translates input queries into triple-based searches on knowledge graphs. Results are later presented in tables and charts. The work of He et al. [49] focused on the proposal of a new graph-based information visualization tool, named ALOHA, to support the identification of relevant information related to dietary supplement.

Classification and search tasks on knowledge graphs have been benefiting from advanced machine learning approaches recently developed. In the work of Sousa et al. [50], for example, the goal was to combine effectively semantic similarity features. The main novelty relied on the use of a genetic programming framework to discovery the best combination function. In the work of Li et al. [51], the focus was on the classification of diseases based on imperfect knowledge graphs, i.e., those that, for instance, lacks enough labeled data to support the creation of training models. Their solution exploited multiple vector representations (e.g., bag-based) and SVM classifiers.

Concerning KGs and AD, Lam et al. [52] converted information from different neuroscience sources to RDF format, making it available as an ontology. AlzPharm [53] used RDF to build a framework that integrates neuroscience information, which also includes Alzheimer, obtained from multiple domains. The goal was to unify the neuroscientists’ queries into a single ontology.

The method proposed in this work differs from the related work in some aspects. We propose a preprocessing of the unstructured text, to identify co-references and abbreviations. It is important to remove such elements from sentences, in a way to refrain from having, for instance, pronouns and abbreviations on the graphs vertices. We also propose in the preprocessing, the simplification of long sentences, where long sentences are replaced by at least two smaller and more cohesive sentences, to ease the work of extracting relations and entities from them. We propose the use of the SRL technique to obtain the main relations (those related to the main verbs of the sentences), in conjunction to a technique based on the dependency parsing output, to obtain secondary relations (related to the nouns of the sentence). For obtaining ontology links, we propose a method that combines named entities recognition through UMLS concepts, and SPARQL queries that find mappings from UMLS to the targeted ontology.

The remainder of this paper is organized as follows: “Methods” section describes and formalizes our proposed solution; “Results” section reports on the experimental evaluations conducted for assessing our proposal whereas “Discussion” section discusses the obtained findings. Finally, “Conclusion” section presents the conclusions and future work.

## Methods

This section describes our proposal for generating ontology-linked KGs from unstructured texts, obtained from the biomedical scientific literature.

In formal terms, a Knowledge Graph $\mathcal {KG} = (\mathcal {V}, \mathcal {E})$ can be represented as a regular graph, containing a set of Vertices $\mathcal {V}$ and Edges $\mathcal {E}$. The vertices express entities or concepts, and the edges express how such concepts and entities relate to each other.

A RDF triple refers to a data entity composed of a subject (*s*), predicate (*p*) and an object (*o*), represented as $t = (s, p, o)$. In KGs, the edges are, then, a set of predicates, such that $\mathcal {E} = \{p_0, p_1, ..., p_n\}$. The vertices are, in turn, a set of subjects and objects, such that $\mathcal {V} = \{s_0, s_1, ..., s_n, o_0, o_1, ..., o_n\}$. In this work, a KG is represented as a set of RDF triples, such that, $\mathcal {KG} = \{t_0, t_1, ..., t_n\}$, where $t_0 = (s_0, p_0, o_0), t_1 = (s_1, p_1, o_1), ..., t_n = (s_n, p_n, o_n)$.

An ontology describes a real-world domain in terms of concepts, attributes, relationships and axioms [54]. Formally, an ontology $\mathcal {O}$ is represented as a set of classes $\mathcal {C}_\mathcal {O}$ interrelated by directed relations $\mathcal {R}$, and a set of attributes $\mathcal {A}_\mathcal {O}$, i.e., $\mathcal {O} = (\mathcal {C}_\mathcal {O}, \mathcal {R}_\mathcal {O}, \mathcal {A}_\mathcal {O})$.

In this sense, we may consider an ontology-linked knowledge graph $\mathcal {KG}^\prime = (\mathcal {V}^\prime , \mathcal {E}^\prime ) = \{t^{\prime }_0, t^{\prime }_1, ..., t^{\prime }_n\}$, having some of its constituents as instances of classes, relations, and attributes of a given ontology $\mathcal {O}^\prime = (\mathcal {C}_\mathcal {O^\prime }, \mathcal {R}_\mathcal {O^\prime }, \mathcal {A}_\mathcal {O^\prime })$. A given predicate $p^\prime \in \mathcal {E}^\prime$ may be an instance of a relation $r^\prime \in \mathcal {R}_{O^\prime }$. A given subject $s^\prime \in \mathcal {V}^\prime$ and an object $o^\prime \in \mathcal {V}^\prime$ may be instances of, either a class $c^\prime \in \mathcal {C}_\mathcal {O^\prime }$, or an attribute $a^\prime \in \mathcal {A}_{\mathcal {O^\prime }}$.

**Fig. 1:**
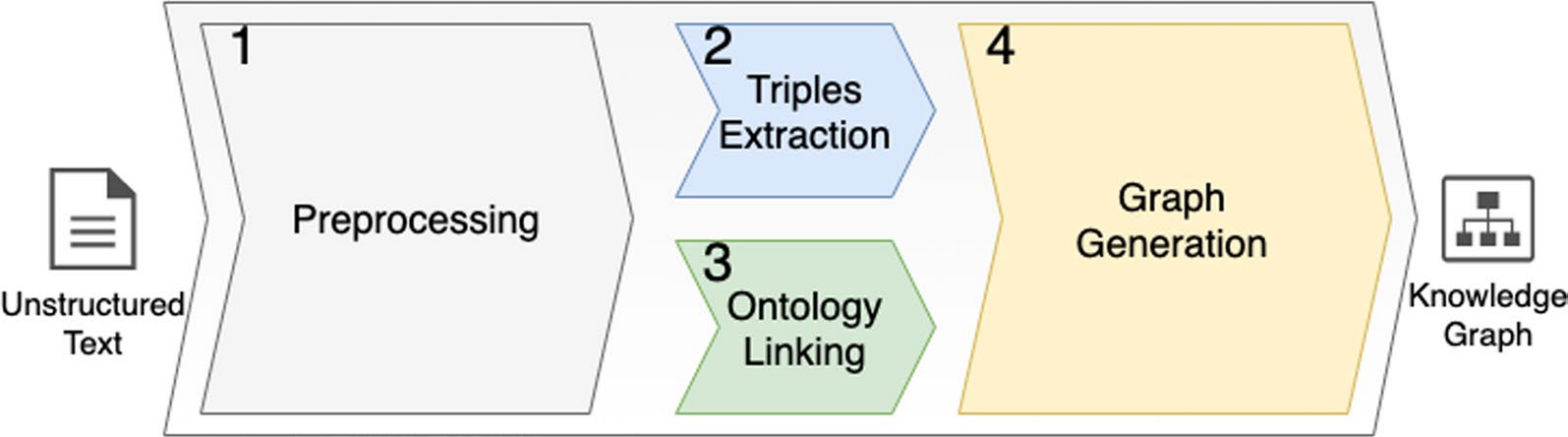
KGen (knowledge graph generation) pipeline. The unstructured text (input) goes through four key steps. An ontology-linked knowledge graph is generated at the end

We introduce our *KGen* (a shorthand for *Knowledge Graph Generation*) method and tool implementation to generate ontology-linked KGs. Figure 1 presents the key components in KGen represented in a pipeline, subdivided into steps. Each step performs modular tasks.

We describe in details each step of the pipeline in the upcoming subsections. Implemented Tool subsection describes the implementation and architectural aspects of the developed KGen software tool.

**Fig. 2:**
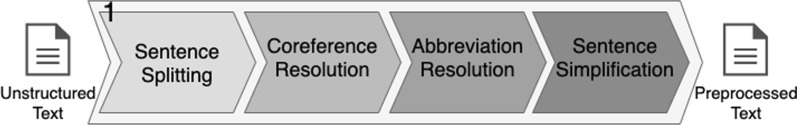
The first key step: preprocessing. The unstructured text (input) goes through four sub-steps. A preprocessed text is generated as output

### Preprocessing

In the preprocessing step, the unstructured text goes through four sub-steps, as shown in Fig. 2, before generating the preprocessed output.

The initial sub-step is the sentence splitting, where sentences are identified from the unstructured input text. Let us consider the input text (*I*) as a set of tokens (*t*), i.e., $I = \{tk_0, tk_1, ..., tk_n\}$. In this context, we consider tokens as words, punctuation, numbers, and other special characters (e.g., parentheses). The input text is also represented as a set of sentences (*s*), i.e., $I = \{s_0, s_1, ..., s_m\}$, in a way that each sentence is a subset of tokens from the input text (e.g., $s_0 = \{tk_0, tk_1, tk_2\}, s_1 = \{tk_3, tk_4, tk_5, tk_6\}, ..., s_m = \{tk_{n-2}, tk_{n-1}, tk_n\})$. The sentence splitting determines how many sentences exist in the input text, and which tokens, from the whole input text set belong to each sentence.

This subtask is performed using two NLP tools. The first one is a tokenizer, which breaks the entire text into tokens. Then, the second tool, a sentence splitter, identifies where sentences begin and end through specific tokens, such as punctuation. Of course, not all punctuation marks indicate the ending of a sentence (e.g., in an abbreviation). For such reason, sentence splitters are often implemented considering a set of rules, or even trained models obtained from huge amounts of texts where sentence boundaries are usually annotated manually. Tokenizers and sentence splitters are available in the most common NLP toolkits (e.g., Stanford CoreNLP [23] and NLTK [55]).

The second sub-step identifies and resolves co-references in the text. Let us take the set of sentences obtained, and the tokens that constitute such sequences. A co-reference is determined when a token (e.g., a pronoun) refers to other token, or set of tokens, in the same, or another sentence. Consider the following two sentences: *This study confirms the high prevalence of poststroke cognitive impairment in diverse populations. It also highlights common risk factors.* The first token in the second sentence, $\{It\}$, is a clear co-reference to the following subset of tokens in the first sentence: $\{This, study\}$. Our technique replaces the first token in the second sentence by the identified subset of tokens, resulting in: *This study confirms the high prevalence of poststroke cognitive impairment in diverse populations*. ***This study*** *also highlights common risk factors.*

Co-reference resolution is performed by observing sets of rules that consider the Parts of Speech of the tokens, as well as syntactical and lexical patterns. Similarly to tokenizers and sentence splitters, the most common NLP toolkits provide co-references resolution utilities.

The third sub-step identifies and resolves abbreviations. Like the co-references resolution, abbreviations can be identified by observing specific subsets of sequential tokens within the sentences. A token (or sequence of tokens) representing an entity, followed by another sequence of tokens that begins and ends with parentheses can determine the introduction of an abbreviation in a text.

In this sense, considering a sentence $s = \{...$, $tk_{e0}$, $tk_{e1}$, ..., $tk_{en}$, (, $tk_{abbr}$, ), $... \}$, one may notice that $tk_{abbr}$ can be determined as an abbreviation, being delimited by two specific tokens representing parentheses, and because either the preceding token $t_{en}$, or the preceding finite sequence of tokens $t_{e0}, t_{e1}, ..., t_{en}$ represents an entity. For instance, consider the following two sentences: *This study confirms the high prevalence of poststroke cognitive impairment (PSCI) in diverse populations. Prevention strategies are required to reduce the prevalence of PSCI.* Clearly, *PSCI* is an abbreviation of *poststroke cognitive impairment*.

Abbreviation identification, therefore, is achieved by observing patterns over sequences of tokens. Once an abbreviation is identified, it is resolved in the text by changing the abbreviation occurrences by the referred term or expression (e.g., *This study confirms the high prevalence of poststroke cognitive impairment in diverse populations. Prevention strategies are required to reduce the prevalence of* ***poststroke cognitive impairment***). Patterns over sequences of tokens are able to be recognized by NLP tools such as Stanford’s *TokensRegex* [56]. Other tools, such as *ScispaCy* [57], provide an abbreviation detection utility for biomedical terms.

The last sub-step is sentence simplification. A common sentence can be subdivided into phrases (comprised of subsets of the sentence tokens), i.e., $s = \{p_0, p_1, ..., p_n \}$, where $p_0 = \{tk_0, tk_1, ..., tk_n\}, p_1 = \{tk_{n+1}, tk_{n+2}, ..., tk_{n+m}\}$, and so on. Typically, complex sentences are composed of several phrases, that may start with a noun phrase, followed by verb phrases. Such phrases may, in turn, be subdivided into other phrases (e.g., prepositional phrases, further verb phrases, and even other noun phrases). Such phrases are commonly bound by conjunctions (e.g., and, but, or, nor, etc.). The job of the sentence simplification sub-step is to detect conjunctions and phrases boundaries and to derive smaller sentences, e.g., $s^\prime = \{p_0, p_1\}$, $s^{\prime \prime } = \{p_0, p1\}$.

**Fig. 3:**
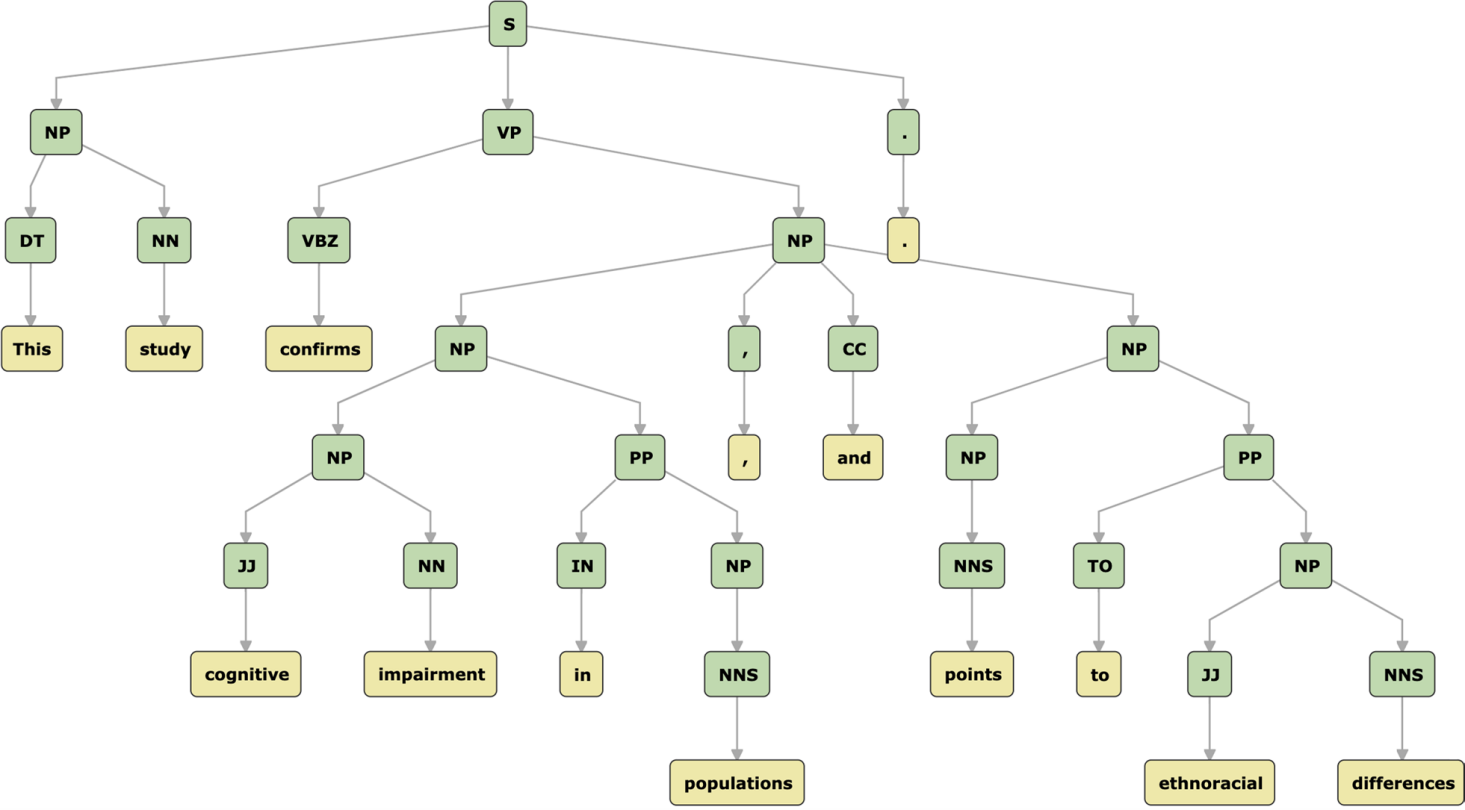
A parse tree. Tokens are the seen at bottom (leaves), with their corresponding parts of speech right above. The root level denotes the sentence, and the intermediary levels denote the phrases

Determining phrases and conjunctions in a sentence is a task achieved with the assistance of a NLP technique called constituency parsing. Figure 3 shows a constituency parsing output (a parse tree) for the following sentence: *This study confirms cognitive impairment in populations, and points to ethnoracial differences.*

In a parse tree, the leaves denote the tokens and their parts of speech. The root of the tree denotes the sentence, and the intermediary nodes denote the phrases. Figure 3 presents that there is a verb phrase subdivided into two other verb phrases, bound by a conjunction (*and*). Such construction is commonly found in long sentences on scientific texts, that could be reduced to smaller, and more cohesive sentences. For the given example, the long sentence is changed into two smaller sentences: *This study confirms poststroke cognitive impairment in diverse populations. This study points to ethnoracial differences.*

**Fig. 4:**
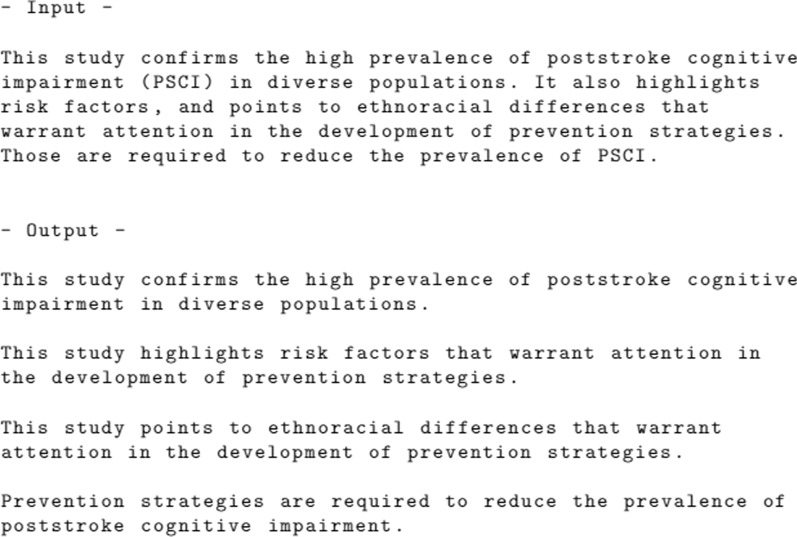
Preprocessing step’s input and output

In summary, the preprocessing step identifies sentences, resolves co-references, resolves abbreviations, and simplifies long sentences. Figure 4 shows an example of an unstructured text input, and the resulting preprocessed output.

### Triples extraction

**Fig. 5:**
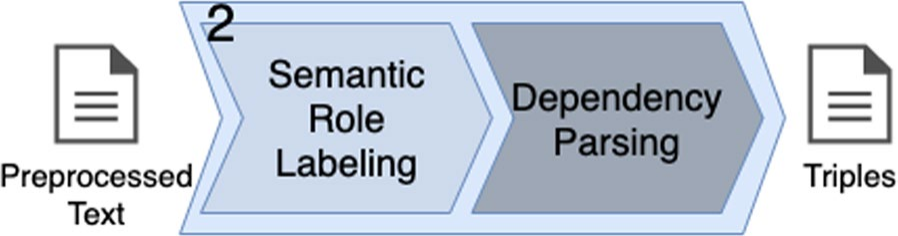
The second key step: triples extraction. The preprocessed text (input) goes through two sub-steps, generating a set of triples as output

With the preprocessed text ready, the next key step in the method consists of extraction of triples. The input text goes through two sub-steps (*cf.* Fig. 5), outputting a set of triples.

Consider a simple sentence $s = \{np, vp\}$, composed of a noun phrase and a verb phrase. The verb phrase in turn, may be composed of a verb *v*, (the main verb of the sentence) and another noun phrase, i.e., $vp = \{v, np^\prime \}$. Such verb in the verb phrase denotes a relation between both noun phrases, considered the verb arguments. This relation can be represented by a triple $t = (s, p, o)$, where the predicate refers to the verb, the subject is the first noun phrase, and the object, the second noun phrase, i.e., $t = (np, v, np^\prime )$. This triple denotes the main relation of the sentence.

Besides the main relation, our technique is suited to extract secondary relations from both noun phrases, which are, ultimately, sequences of tokens. A noun phrase is typically composed of a set of tokens, denoting, for instance, nouns, adjectives, and determiners. From such compositions, it is possible to derive secondary relations. The triples extraction step in our method requires two sub-steps: the first for extracting the main relations, and the second one for extracting secondary relations.

In the first sub-step, the SRL technique is applied to identify verbs and their arguments. Consider the following example sentence: *This study confirms the high prevalence of poststroke cognitive impairment.* The verb is *confirm*, and its arguments, per SRL, are *A0: this study; A1: the high prevalence of poststroke cognitive impairment*. From this example, we build the following triple (removing determiners and other preceding stop words):

- (*“study”, “confirms”, “high prevalence of poststroke cognitive impairment”*).

In order to represent the information in a more meaningful manner, the SRL technique considers the retrieval of the role names for the verb arguments, using VerbNet or PropBank resources. Such resources describe sets of roles (rolesets) that were manually put together by linguists. A roleset describes a possible set of roles assumed by the verb arguments, in different contexts. Considering the example, the extracted verb and its arguments, SRL obtains the *confirm.01* roleset as a match, which describes as role names *A0:Agent, A1:Theme*. In this way, we may use a different reification form, to represent the information in two triples:

- (*“confirms”, role:Agent, “study”*)
- (*“confirms”, role:Theme, “high prevalence of poststroke cognitive impairment”*)

**Fig. 6:**
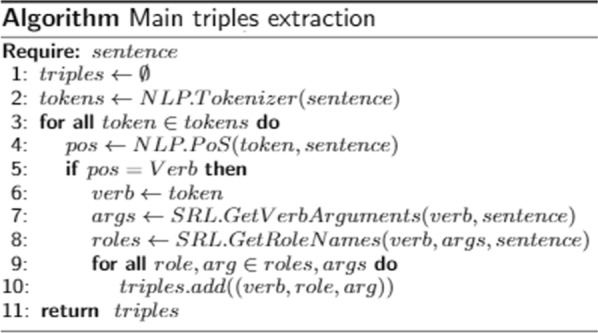
Algorithm for extracting the main triples

The predicates in such triples are *Universal Resource Identifiers* (URIs), instead of literals. They are defined locally, and represent the role types. Figure 6 summarizes the designed procedure.

**Fig. 7:**
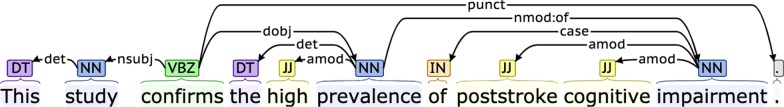
Dependency parsing output. At the bottom are the sentence tokens, with their corresponding parts of speech on top. The arrows show the labeled dependencies between the tokens

In the next sub-step, an NLP technique called dependency parsing is performed in the sentence to determine secondary triples. The dependency parsing output is represented in a tree form (cf. Fig. 7).

The PoS tags are shown right above the tokens, whereas the dependencies are linked by types, through arrows. We observe that the nouns may have modifiers (usually adjectives linked by modifier types), e.g., *prevalence* has the *high* modifier; *impairment* has both *cognitive* and *poststroke* modifiers. From such information, we derive rules to build secondary triples. For instance, we consider some as sub-classes of others:

- (*“high prevalence”, rdfs:subClassOf, “prevalence”*)
- (*“cognitive impairment”, rdfs:subClassOf, “impairment”*)
- (*“poststroke cognitive impairment”, rdfs:subClassOf, “cognitive impairment”*)

We notice that there are nouns that modify other nouns, e.g., *poststroke cognitive impairment* modifies (through *of*) *high prevalence*. We consider rules to build triples that represent such information:

- (*“high prevalence of poststroke cognitive impairment in diverse populations”, local:of_poststrokecognitiveimpairment, “high prevalence”*)
- (*“high prevalence of poststroke cognitive impairment in diverse populations”, local:highprevalence_of, “poststroke cognitive impairment”*)

**Fig. 8:**
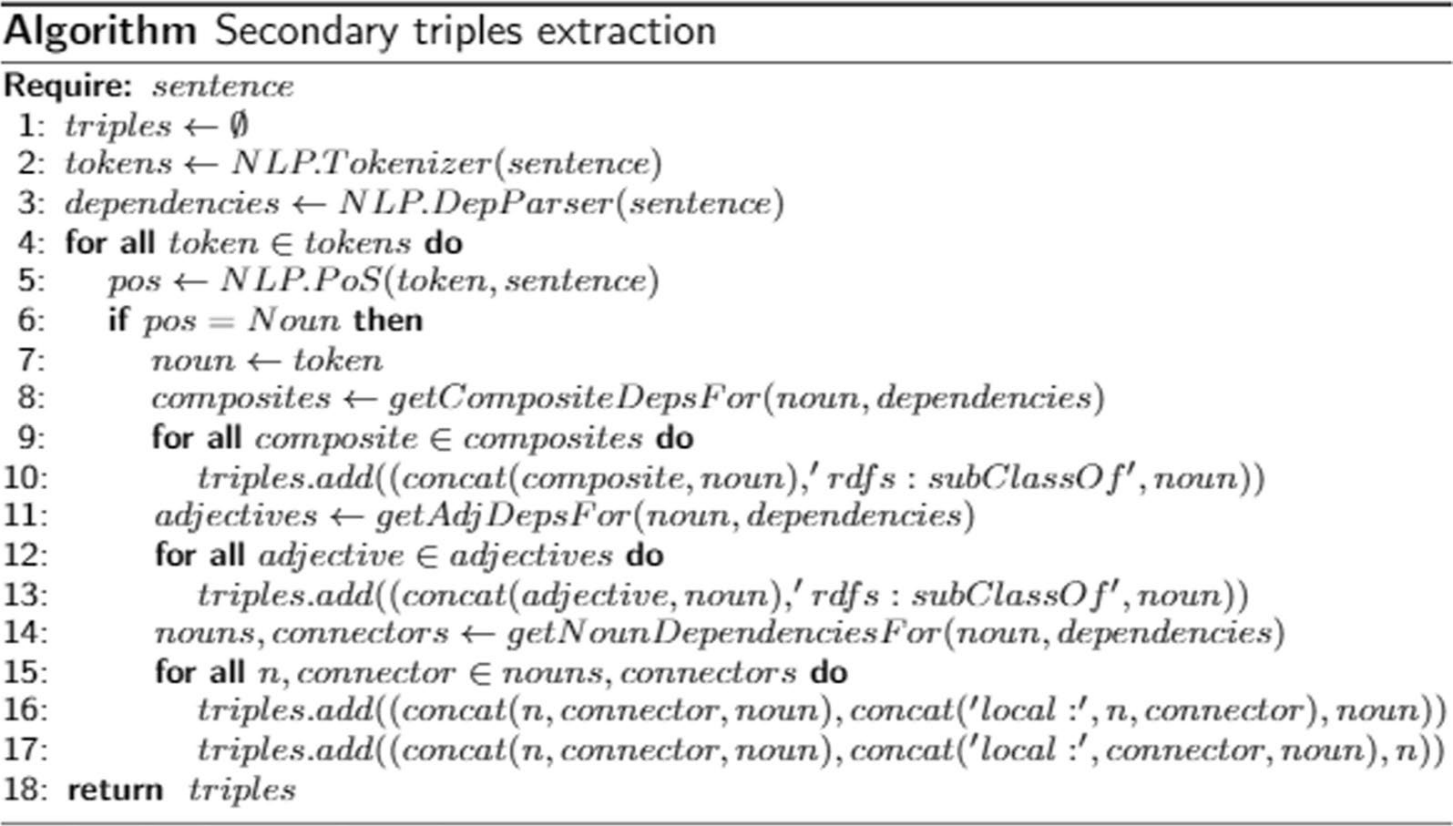
Algorithm for extracting the secondary triples

Figure 8 formalizes the proposed procedure. The predicate in those triples are local resources representing the modifying types. The subjects are the same as the Theme identified in the main triple from the first sub-step, which properly links all the triples.

**Fig. 9:**
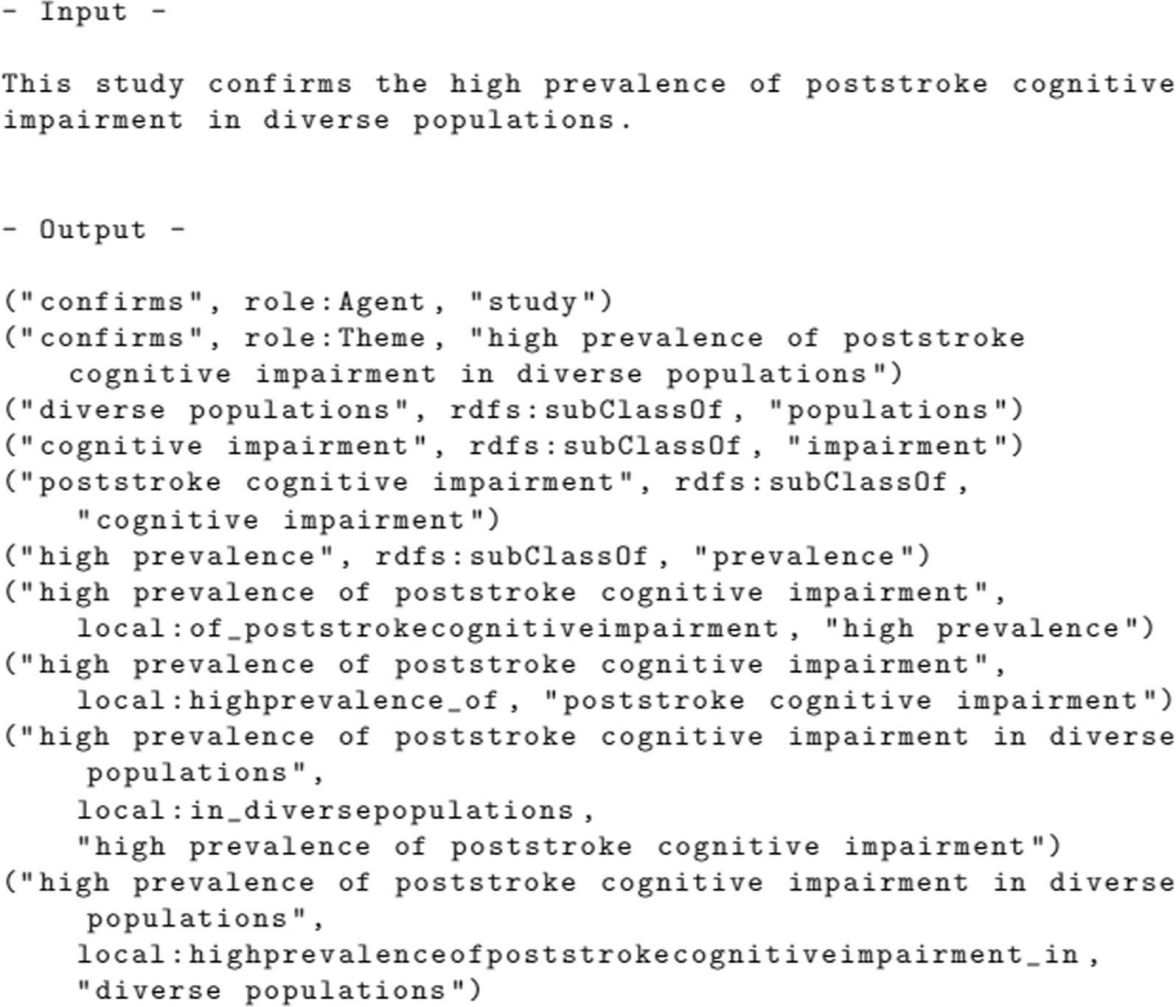
Triples extraction step’s input and output

Figure 9 illustrates the entire step’s output, showing all the triples extracted from a given sentence.

### Ontology linking

**Fig. 10:**
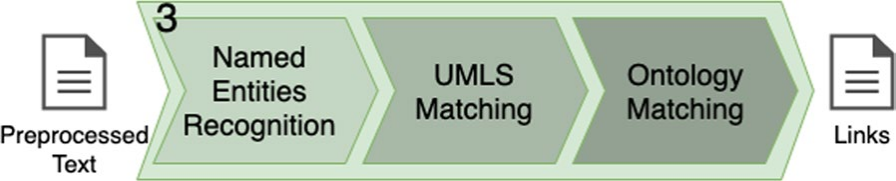
The third key step: ontology linking. The preprocessed text (input) goes through three sub-steps. A set of ontology links are generated as output

This step can be considered optional, as without it, a KG is still generated by the method. For our objective of generating a KG linked to a biomedical ontology, this step plays a key role. It takes as input the preprocessed text, which goes through sub-steps to generate a set of links, as illustrated in Fig. 10.

Entities and relations from a KG may be mapped to classes, properties, and attributes described in a computational ontology. Considering the extracted triples, relations are represented by the predicates, whereas entities are represented in subjects and objects.

Taking as an example a triple $t = (s, p, o)$, and considering $s = \{tk_0, tk_1, tk_2\}$, $p = \{tk_3\}$, and $o = \{tk_4, tk_5\}$. An entity *e* may be represented by subsets of tokens from the subjects or objects. Considering as examples, a possible entity $e_0 = \{tk_4, tk_5\}$, and another $e_1 = \{tk_5\}$, also $e_2 = \{tk_1\}$, and perhaps $e_3 = \{tk_0, tk_1, tk_2\}$. In these examples, entities $e_0$ and $e_3$ correspond to the whole object *o* and the whole subject *s* of the triple *t*. Entities $e_1$, and $e_2$, on the other hand, correspond to parts of *s* and *o*

In this context, it is important to identify the named entities and the relations in the text, and find their respective links (i.e., classes, properties, or attributes) in target ontologies, for later matching such links to portions of the KG (as further described in “Graph generation” section).

The first sub-step is the recognition of named entities in the given sentences, using a NER technique. Considering the usual example sentence (*This study confirms the high prevalence of poststroke cognitive impairment in diverse populations*), the following are examples of recognized named entities: *study*, *prevalence*, *impairment*, *cognitive impairment*, *populations*.

NER requires models that are trained to find suitable entities. Since we are working under the biomedical domain, it requires a model that has been properly trained to find biomedical entities. Such models are available, for instance, in the *ScispaCy* [57] library, as adopted in this investigation.

The following sub-step aims at matching the named entities and relations to UMLS. Relations are identified by searching for verbs, using a PoS tagger, and taking their lemmatized form. The *ScispaCy* library, besides identifying named entities, performs the matching of such entities and relations to a database containing UMLS’ Concept Unique Identifiers (CUIs), along with a UMLS description of the matched entries.

**Fig. 11:**
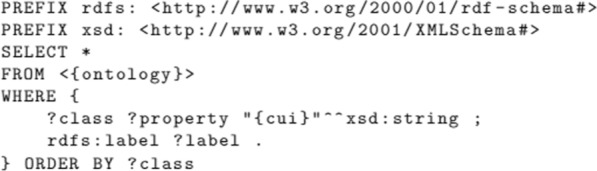
SPARQL query example for mapping UMLS CUIs to the final ontology

As we have all the CUIs for the UMLS-matched terms, we look for matches in a targeted biomedical ontology in the last sub-step. We retrieve the final ontology matching by performing SPARQL queries, similar to the template presented in Fig. 11. Such query finds classes containing the UMLS’ CUIs to ultimately link the named entities to a biomedical ontology.

**Fig. 12:**
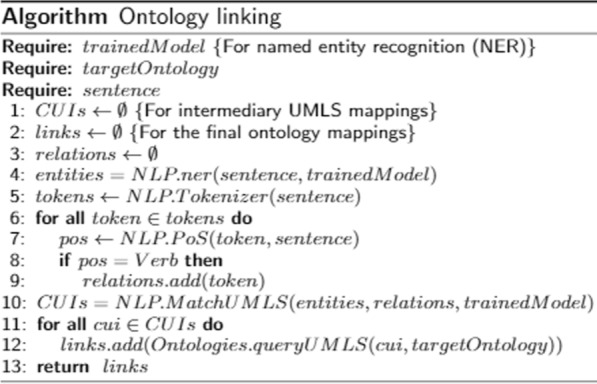
Algorithm for ontology linking

Figure 12 summarizes the whole ontology linking procedure. The trained model for NER technique and the target ontology are excepted as input for the algorithm. The algorithm generates a set of links, which are added in the KG.

**Fig. 13:**
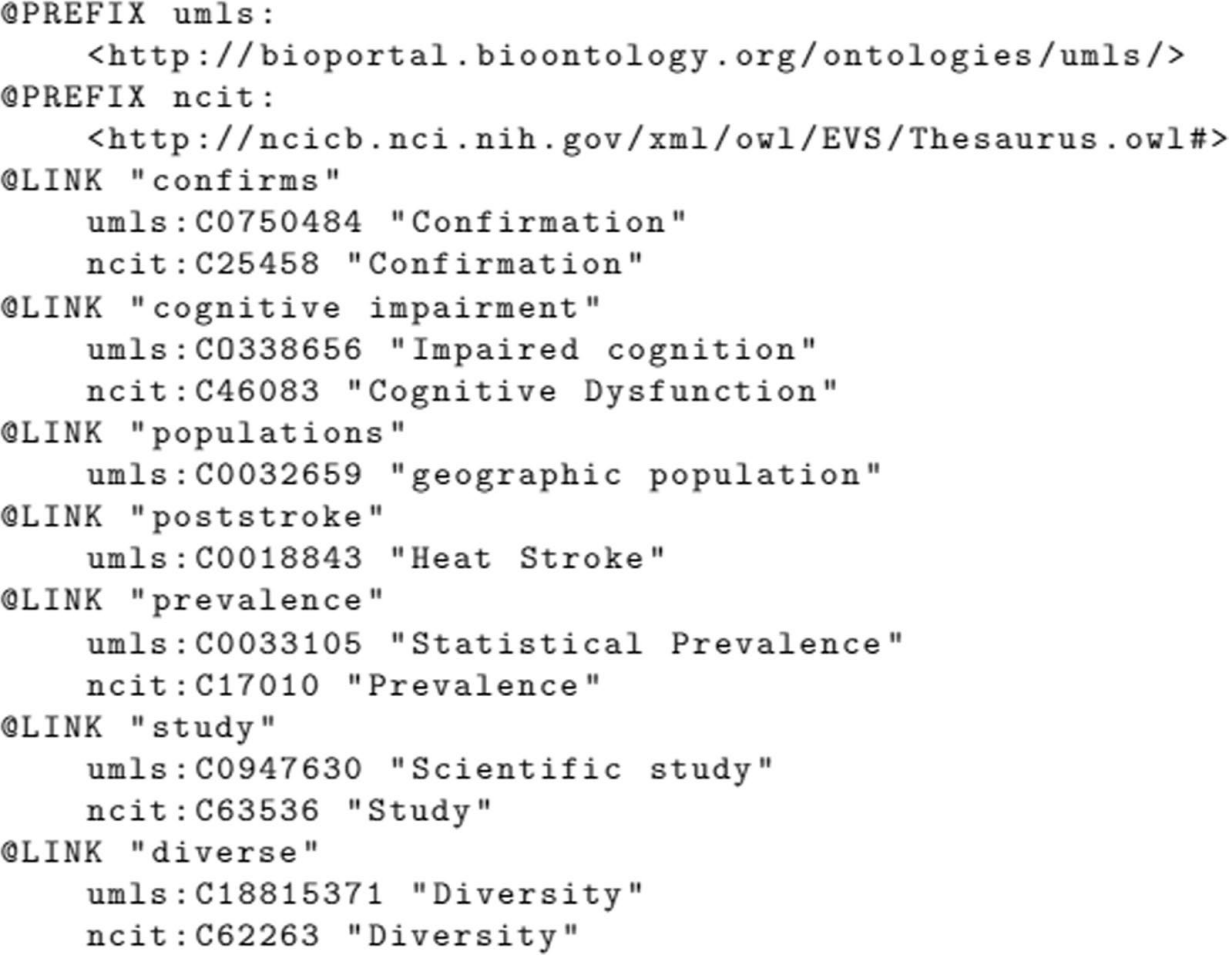
Ontology linking step’s output

Figure 13 shows examples of entities/verbs identified, with intermediary UMLS mappings, and final mappings for the National Cancer Institute Thesaurus (NCIT) ontology [58]. We chose to use NCIT as the target ontology in this example, as it is the ontology suggested by NCBO recommender service [59] for the used textual excerpt.

### Graph generation

**Fig. 14:**
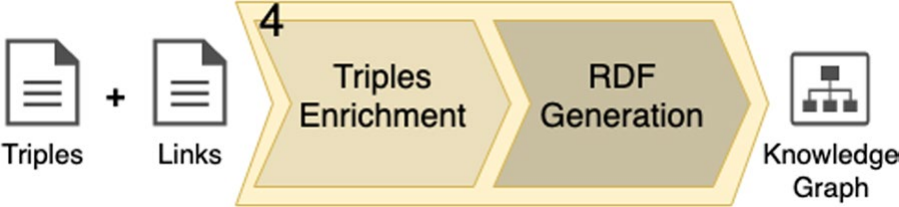
The final key step: graph generation. The sets of triples and links (inputs) go through two sub-steps before generating an ontology-linked knowledge graph as output

The final step in our method takes as input the set of triples (generated in step 2), and the set of links (generated in step 3). Such inputs go through two sub-steps to generate an ontology-linked KG. Figure 14 illustrates the expected inputs and outputs of this final step.

The first sub-step is the triple enrichment. This sub-step performs tasks to combine the extracted triples and generate a KG. We create local URIs for all strings in the set of triples’ subjects and objects. Such resources are linked with its respective literal representations through new triples, using the *rdfs:label* predicate. The local URIs are meant to generate a fully-connected graph, as much as possible, as we may reuse local URIs on different triples. New triples are created to bind the ontology mappings to their respective named entities and relations in the graph, through the *owl:sameas* predicate.

Finally, with the new set of triples generated in the previous sub-step, a content is generated in Terse RDF Triple Language (Turtle) format. This is the final output of the entire pipeline. Optionally, graphical representations may be generated from this Turtle file, mapping the triples constituents to edges (predicates) and vertices (objects and subjects).

**Fig. 15:**
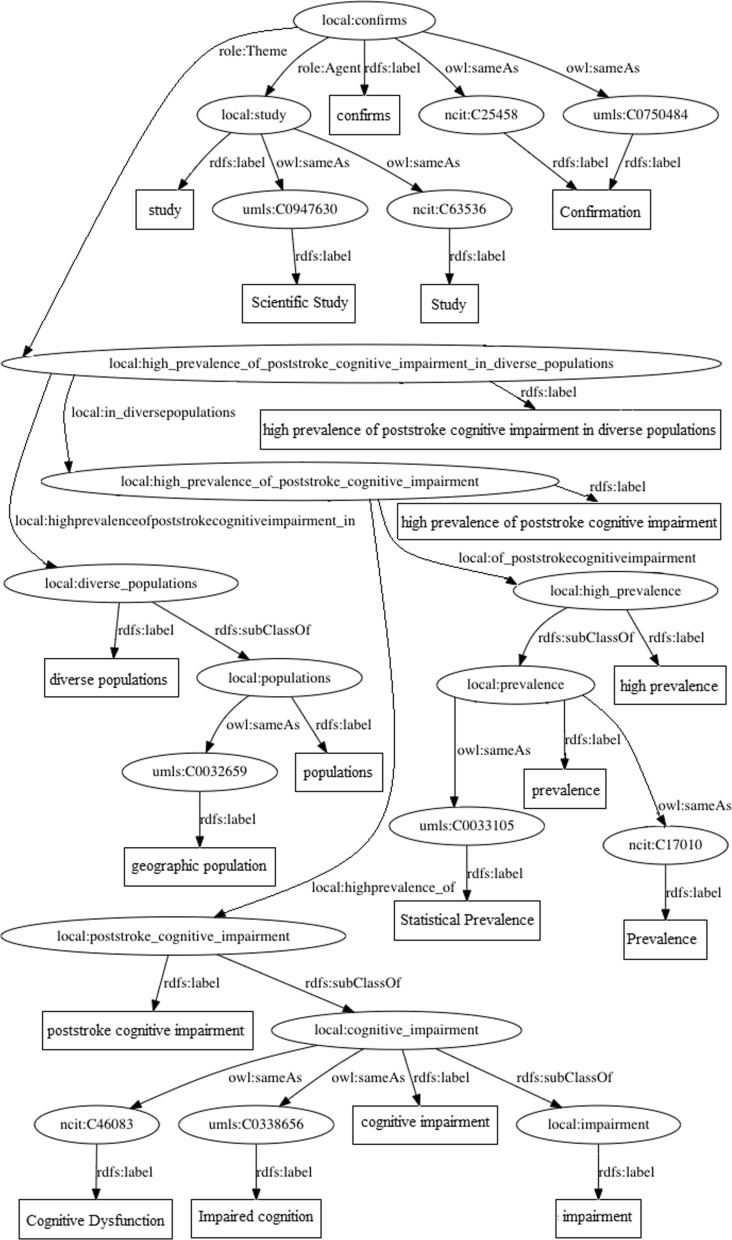
Graphical representation. Ontology-linked knowledge graph generated from the following sentence: *This study confirms the high prevalence of poststroke cognitive impairment in diverse populations.*

Due to the amount of triples, such representation may be a bit overwhelming to explore. Figure 15 exemplifies this by presenting the graphical representation of the output generated for the following sentence: *This study confirms the high prevalence of poststroke cognitive impairment in diverse populations.*

In Fig. 15, we observe the main relation extracted from the SRL technique output at the top of the figure. URIs are represented by ellipses, whereas literals are represented by rectangles. The edges represent the predicates of the turtle file’s triples. Secondary relations obtained from the dependency parsing were derived from the main relation. Intermediary UMLS mappings and final NCIT mappings are also presented, represented by the nodes with the *umls* and *ncit* prefixes.

**Fig. 16:**
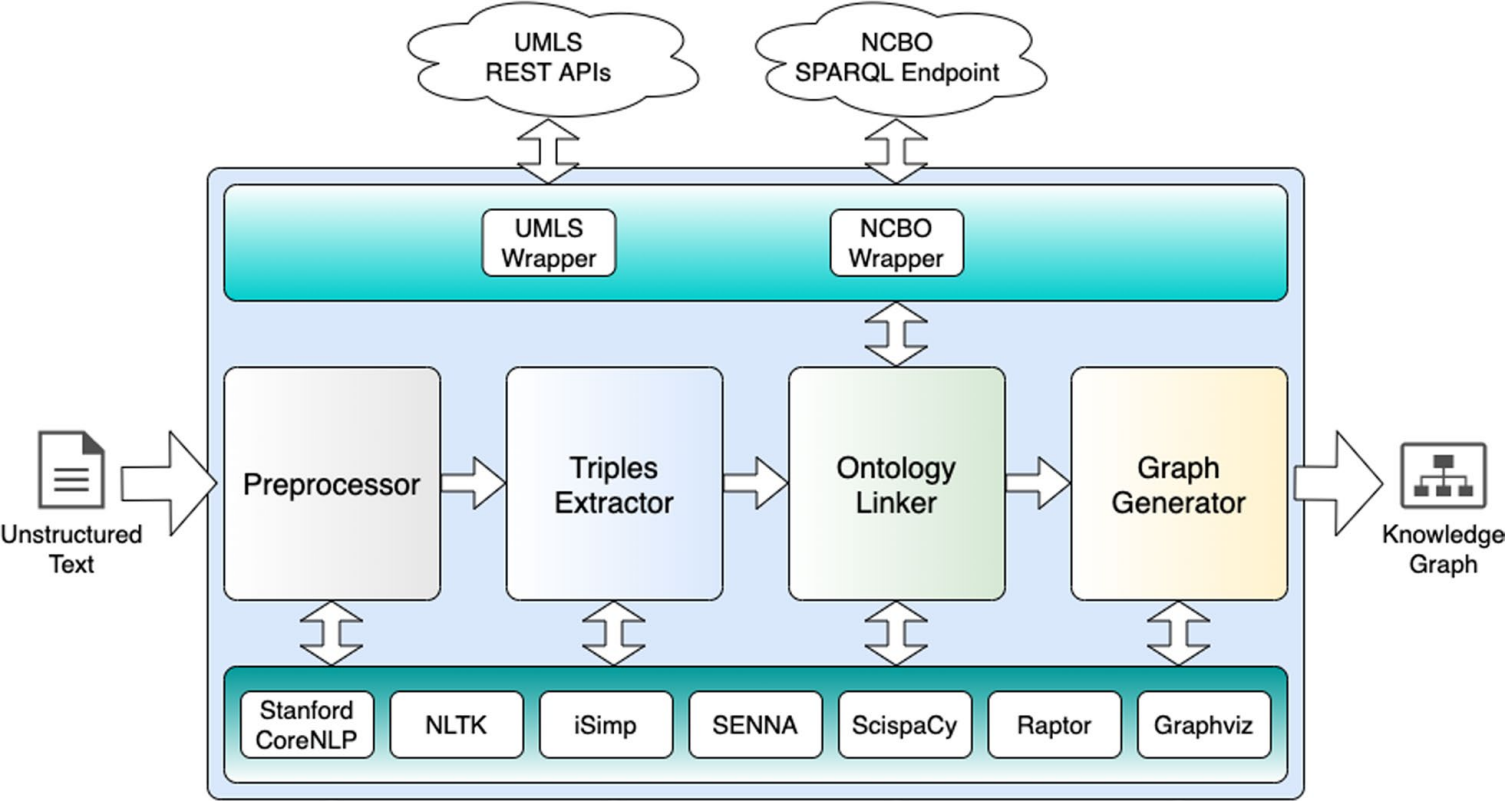
Implemented tool architecture. The four key KGen steps are implemented in four components, seen at the central portion. In the lower portion there are 3rd party components. In the upper portion, there are wrappers for external services

### Implemented tool

This subsection describes resources and tools used in the implementation of a KGen tool, fully available at https://github.com/rossanez/kgen (As of Jan. 2020), using Python language. The implementation was a mean to validate our method. Figure 16 illustrates the tool’s architecture. The four key steps from the method (i.e., preprocessing, triples extraction, ontology linking, and graph generation) are implemented in four components. All the third party tools/toolkits used in the components implementation are represented at bottom of the figure, in a common layer that can be accessed by all components. Finally, the external services are represented at top. This is another common layer that contains wrappers used to intermediate the communication between the main components and the external services.

In the main components, Stanford CoreNLP [23] is used for sentence splitting, co-reference resolution, tokenizing, PoS tagging, constituency parsing, and dependency parsing. Since it is a Java toolkit, a server was implemented in Python, to process such NLP tools requests and return the results. The default models for English language were used in all tools, except for the co-reference resolution. In this case, we chose Stanford’s neural co-reference model. It runs slower than the default model, but it presents better results for texts written in English language [23]. We chose accuracy over running speed in this posed trade-off.

In the preprocessor component, the sentence simplification uses *iSimp* [60], a sentence simplification system that relies on models trained specifically for biomedical texts. Abbreviation identification is performed using ScispaCy tool [57], which implements a detection algorithm proposed by Schwartz and Hearst [61]. As ScispaCy outputs a list of identified abbreviations, an additional implementation based on NLP tools is used to replace the identified abbreviations by their complete form throughout the whole text.

In the extraction of triples, *SENNA* [33] system is used to perform the SRL technique. *SENNA* has been chosen as it shows good accuracy in texts from the biomedical domain [62]. We used NLTK’s VerbNet and PropBank corpus readers interfaces to determine the roleset and the verb argument role names. Lemmatization is performed using NLTK.

In the ontology linker, ScispaCy [57] models are used in conjunction with NLTK to determine named entities, and to obtain UMLS CUIs. ScispaCy provides several models that are meant to adapt NLTK in processing biomedical text. For this implementation, we chose the larger biomedical vocabulary available (As of Nov. 2019), encoded in 600K word vectors for the English language, namely, ScispaCy’s *en_core_sci_lg* model, enabling more biomedical entities to be recognized than the other available models. Once UMLS CUIs are obtained, we submit SPARQL queries to the NCBO SPARQL endpoint [22] to obtain the final ontology mappings.

In the graph generator, the conversion of the turtle file contents to graphs edges and vertices are performed using *Raptor* [63]. The graph image is generated from the set of edges and vertices using *Graphviz* [64].

## Results

An evaluation was conducted to assure the quality of the KGs generated through our solution. To this matter, we conducted two experiments. Our objective is to guarantee that the key steps from our method produce the most appropriate outputs. We aim to ensure that extracted triples from the sentences are similar to triples that would be manually extracted by domain specialists, and ultimately, ensure that generated KGs make sense to specialists. In addition, we assess to which extent the proposed linking method improves the final output of our method.

The first experiment (cf. Experiment I: Evaluation of Triples subsection) involved two physicians who kindly volunteered to assist us in evaluating the quality of the triples extracted from biomedical texts. To the best of our knowledge, there are no gold standards in literature to evaluate triples extracted from unstructured texts from the biomedical domain. In this way, we have chosen to invite subjects that are knowledgeable in this domain for this experiment. Both physicians have more than 10 years of working experience in their areas, and they attend international events regularly, thus, are used to read scientific papers from the biomedical domain. As a disclaimer, it is important to mention that, at any time preceding the experiment, neither of the physicians were told about the actual nature of this work, or what does our method achieves.

The second experiment (cf. Experiment II: Ontology links subsection) involved a comparison of the ontology links obtained from the current version of our method, in comparison with the initial version, as described in the work by Rossanez and Dos Reis [14]. With this experiment, we assess the difference between using a trained model to recognize biomedical named entities and their UMLS CUIs, to ultimately link with a final biomedical ontology, against using NCBO’s REST APIs to provide the final biomedical ontology links directly.

### Experiment I: evaluation of triples

This experiment, as previously stated, involved two physicians, and consisted of two parts. The preamble of the experiment was to have each physician read three distinct abstracts (i.e., six distinct abstracts in total), extracted from medical papers related to Alzheimer’s Disease, from the *Neurology* journal. The abstracts of this journal follow the Objective, Methods, Results, and Conclusions format. We took the six first publications which resulted from a search for the *Alzheimer’s Disease* term, in the journal’s search engine, at https://n.neurology.org (As of Dec. 2019).

#### Extraction of triples analysis

A small introduction about RDF was given to the subjects in the first part of the experiment. We presented some examples of RDF triples (subject, predicate, and object) extracted from small sentences. We made sure that they completely understood the process before starting the main procedure of the experiment.

We asked them to only take into account the conclusion section of the abstracts to manually create their own triples (graph) according to their interpretation of the text. Such sub-section contains, in general, one or two small sentences (e.g., This study confirms the high prevalence of poststroke cognitive impairment in diverse populations, highlights common risk factors, in particular, diabetes mellitus, and points to ethnoracial differences that warrant attention in the development of prevention strategies.). No communication was allowed between the subjects or the subjects and the experimentalist, until the process was finished.

We ran our tool, which implements our KGen method, to extract triples from the same abstracts’ conclusions sub-sections handed to the subjects. Table 1 summarizes the amount of triples extracted from the 6 abstracts, labeled from *A00* to *A05*, by the subjects, and also, by our method.

**Table 1:** Comparison of extracted triples between experts and KGen results

	A00	A01	A02	A03	A04	A05
Experts	9	7	12	7	10	5
KGen	32	38	62	65	59	80

Amount of triples extracted by the domain experts, and by KGen, for each abstract, labeled from *A00* to *A05*

As shown by Table 1, our method has extracted more triples than the subjects. This is achieved due to the amount of main and secondary relations that our method extracts from the sentences. The secondary relations are extracted using the dependency parsing technique, resulting in triples that relate nouns with their compounds and adjectives, as well as other nouns. This may result in a great amount of triples, depending on the amount of these parts of speech in the sentence. For instance, consider the following sentence: *Ethnoracial differences warrant attention in the development of prevention strategies*. An example of manually extracted triple by one of the experts is the following:

- (*“Ethnoracial differences”, “warrant”, “attention in the development of prevention strategies”*)

On the other hand, using our tool, the dependency parsing technique resulted in the following set of secondary triples:

- (*“prevention strategies”, rdfs:subClassOf, “strategies”*)
- (*“ethnoracial differences”, rdfs:subClassOf, “differences”*)
- (*“development of prevention strategies”, local:of_preventionstrategies, “development”*)
- (*“development of prevention strategies”, local:development_of, “prevention strategies”*)

As for the main relations, extracted using the SRL technique, they result in at least two triples for sentence, depending on the number of verb arguments that are retrieved by the technique. Considering the same sentence, our tool outputs the following set of triples from the SRL technique:

- (*“warrant”, local:AM-LOC, “development of prevention strategies”*)
- (*“warrant”, vn.role:Agent, “ethnoracial differences”*)
- (*”warrant”, vn.role:Theme, “attention”*)

The predicate of the first triple is an URI that indicates location. The predicates from the second and third triples are URIs representing the role that the object assumes in the original sentence. If we consider the SRL technique alone, without the proposed reification form, then we have the following triples, that are in a format that is similar to the manually extracted by the experts:

- (*“ethnoracial differences”, “warrant”, “attention”*)
- (*“ethnoracial differences”, “warrant attention in”, “development of prevention strategies”*)

For a fair base of comparison, we ran our tool considering different configurations for triples extraction: (1) Extracting only the main relations through the SRL technique; (2) Extracting only the main relations through the SRL technique, without considering the adopted reification form; And (3) extracting only the secondary relations through the dependency parsing technique. Table 2 summarizes the differences in the results.

**Table 2:** Comparison between KGen’s configurations

	A00	A01	A02	A03	A04	A05
SRL	9	9	15	10	17	11
SRL w/o reification	5	5	9	6	9	6
Dependency parsing	23	29	47	55	42	69

Number of triples extracted considering three distinct KGen configurations: semantic role labeling (SRL) only, SRL without reification form, and dependency parsing only

As another approach to compare KGen’s generated triples with the manually extracted ones, we used the *Jaccard similarity coefficient*. This coefficient measures the similarity between finite sets, and it is defined as the size of the intersection divided by the union of the sets. Equation 1 shows how the Jaccard coefficient *J*(*A*, *B*) is calculated for two sets *A* and *B*.

1 $$\begin{aligned} J(A, B) = \frac{\mid A \cap B \mid }{\mid A \cup B \mid } = \frac{\mid A \cap B \mid }{\mid A \mid + \mid B \mid - \mid A \cap B \mid } \end{aligned}$$

The Jaccard coefficient ranges between 0 and 1. If both sets have the same elements, the value is 1. If there is no intersection between such sets, the value is 0. If both sets are empty, the Jaccard coefficient is defined as 1.

To obtain the Jaccard coefficient, we considered the sets of manually generated triples and KGen’s. From such two sets, we identified triples that were only found in the manual process, triples that were only found by KGen, and finally, triples that are found both manually and by KGen (i.e., the intersection between both sets). Table 3 presents the obtained results.

**Table 3:** Comparison between triple sets

	A00	A01	A02	A03	A04	A05
Manually only	5	5	7	5	7	4
KGen only	28	36	57	63	56	79
Both	4	2	5	2	3	1
*J*(*Man*., *KGen*)	0.10	0.04	0.07	0.02	0.04	0.05

Number of triples found only manually, only by KGen, and by both methods (i.e., the intersection between manual and KGen–this considers triples with the same subject, predicate, and object). Also, the Jaccard similarity coefficient for the sets of triples

We observe that, according to the Jaccard coefficient, the sets are not very similar, having a very little intersection. This is primarily due to the differences between the number of elements from the compared sets, i.e., KGen extracts much more triples than the specialists.

We already discussed that the manually extracted triples are more similar to the triples extracted from the SRL technique without the proposed reification form. In this way, we further compared the KGen triples extracted using the SRL without reification configuration. Such results are described in Table 4.

**Table 4:** Comparison between triple sets (KGen on SRL without reification)

	A00	A01	A02	A03	A04	A05
Manually only	5	5	7	5	7	4
KGen only	1	3	2	4	6	2
Both	4	2	5	2	3	1
*J*(*Man*., *KGen*)	0.40	0.20	0.31	0.22	0.18	0.57

Number of triples found only manually, only by KGen on SRL without the adopted reification form, and by both methods (i.e., the intersection between manual and KGen). Also, the Jaccard similarity coefficient for the sets of triples

The analysis concerning the extraction of triples by KGen using the SRL without reification configuration, the Jaccard coefficient increases. The intersection comparing the amount of triples that were extracted only in the manual and only in KGen’s sets can be explained by some particularities that were found in the manually extraction of triples. One of such is the fact that the experts derived relations that are not explicitly in the sentences. For example, the first abstract contains the following sentence: *This study confirms the high prevalence of poststroke cognitive impairment in diverse populations, highlights common risk factors, in particular, diabetes mellitus, and points to ethnoracial differences that warrant attention in the development of prevention strategies.* Some of the triples the experts were able to extract are:

- (“diabetes mellitus”, “is”, “risk factor”)
- (“poststroke cognitive impairment”, “is prevalent”, “in diverse populations”)
- (“development of preventions strategies”, “are”, “needed”)
- (“prevalence of poststroke cognitive impairment”, “involves”, “ethnoracial differences”)

Although such triples make perfect sense, KGen is not able to build them given the employed techniques. This is mostly due to their predicates not being explicitly found in the text. It requires some logical thinking to build them, and in some cases, even a previous domain knowledge (which is expected from such specialists).

Another example from the following sentence is in the third abstract: *Results at 3 years after unilateral transcranial magnetic resonance-guided focused ultrasound thalamotomy for essential tremor, show continued benefit, and no progressive or delayed complications.* In this case, the specialists were able to distinguish *essential tremor* as the condition, and the (very large) treatment type *unilateral transcranial magnetic resonance-guided focused ultrasound thalamotomy*, resulting in the following triple:

- (“unilateral transcranial magnetic resonance-guided focused ultrasound thalamotomy”, “is”, “an option to manage essential tremor”)

In other cases, some triples were derived from complex sentences. This is handled by KGen in the preprocessing step, ending up on avoiding such redundancies. For example, the following sentence from the second abstract: *High-convexity tight sulci may confound clinical and biomarker interpretation in Alzheimer’s Disease clinical trials.* KGen extracted two triples in this case, whereas the expert extracted the following three triples:

- (“High-convexity tight sulci pattern”, “may confound”, “clinical and biomarker interpretation in Alzheimer’s Disease clinical trials”)
- (“High-convexity tight sulci pattern”, “may confound”, “clinical interpretation in Alzheimer’s Disease clinical trials”)
- (“High-convexity tight sulci pattern”, “may confound”, “biomarker interpretation in Alzheimer’s Disease clinical trials”)

In this sense, we found that such triples represent a secondary knowledge that is derived from the primary knowledge obtained from the text, which is, in turn, represented by the triples extracted by KGen. Therefore, an important lesson learned is that, no matter what technique or method used to extract triples from texts in the biomedical domain, it is important to allow the later addition of manually generated triples from experts to the output. Such semi-automatic approach might enrich the knowledge representation, both in terms of explicit knowledge from the text, but also, in terms of derived knowledge that is rather implicit in the text. Another possibility is to include a new sub-step, or post-processing sub-step, in which we could automatically attempt to derive triples from the obtained triple set, similar to the ones manually obtained from the subjects, through inference reasoning.

#### Knowledge graph analysis

Once the first part of the experiment was finished, we started the preparation for the second part. Both subjects were explained the concept of Knowledge Graphs. They were instructed about the graphical representation of RDF triples in a KG (i.e., edges representing the predicate, while subjects and objects are represented either by ellipses in case of URIs, or by rectangles in case of literals). Once again, we made sure that the concepts were completely understood by them, before moving further.

**Fig. 17:**
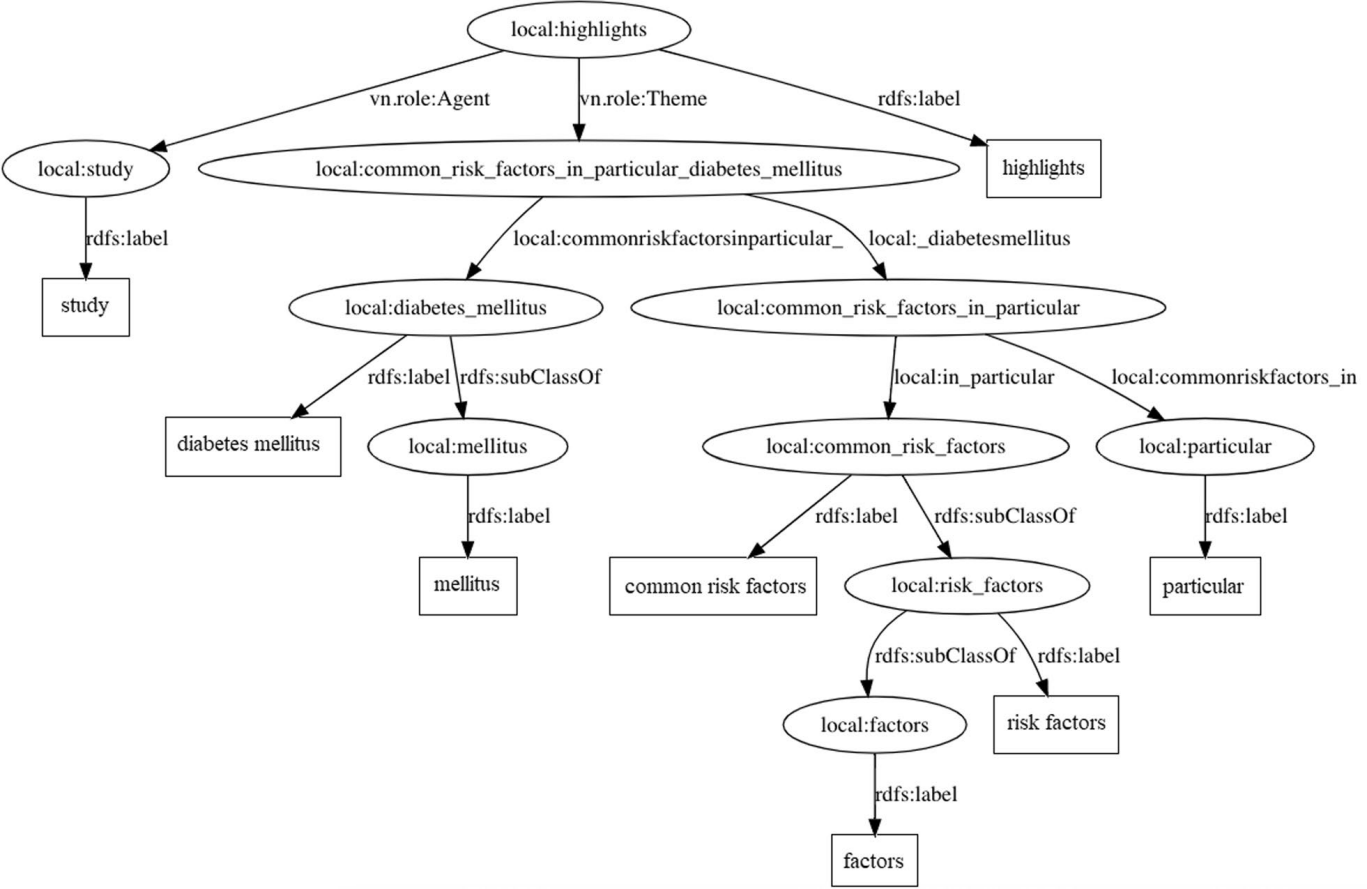
Reduced knowledge graph example. Knowledge graph generated for the triples extracted from the following sentence: *This study highlights common risk factors, in particular diabetes mellitus.*

Each subject was then presented a sentence extracted from one of the abstracts’ conclusions subsection, along with a simple KG generated for that sentence. The presented KG was a simpler version of the KG generated by our method, as it did not present any ontology link. This was meant to remove the extra complexity that such figure may present and become very large. Figure 17 presents one of such graphs.

With those KGs in hand, we asked the subjects to analyze them and freely provide any comments they might have. It is important to mention here that neither of the subjects were told that such KGs were generated by a tool which implements our method, to avoid any kind of bias in their judgements.

Both subjects found such an interesting form to visually describe the sentences from the conclusion subsection of the abstracts. They agreed that the starting point of the graph with the main verb (cf. Fig. 17) is a good starting point as it represents the principal information from the sentence. This reflects the idea of a main relation, represented by the main triples extracted from the SRL technique. This binds the secondary information, represented by the secondary triples extracted from the dependency parsing technique. This binding is well represented by the local URIs that make the graph fully connected.

The subjects agreed that breaking down larger objects and subjects (e.g., *common risk factors* into *risk factors* and then, into *factors*) into smaller and more cohesive terms makes it easier to find a specific concept in the representation. In practical terms, this could allow a SPARQL query performed in the KG on finding if a specific concept is present in the graph. Such task would be harder to accomplish if this concept should be embedded into a graph node that represents a more specific concept (e.g., *common risk factors*), rather than a more generic concept (e.g., *risk factors*).

Still regarding the breakdown of larger nodes into smaller ones, one point of improvement was indicated by the subjects. When, for instance, breaking down *common risk factors* into *risk factors*, and in turn, into *factors*, some terms are left aside, such as *common*, and *risk*. Representing such terms could enrich the knowledge representation, especially because they could also be considered in a SPARQL query. In practical terms, they could be linked to ontologies, as further discussed in Experiment II (*cf*. “Experiment II: Ontology links” section). One possible way to represent such terms, would be to add new triples to the existing set, using local URIs that would represent those as part of the initial specific concept, such as the ones represented in bold below:

- (“common risk factors”, rdfs:subClassOf, “risk factors”)
- **(“common risk factors”, local:hasAdjective, “common”)**
- (“risk factors”, rdfs:subClassOf, “factors”)
- **(“risk factors”, local:hasCompound, “risk”)**

Another point of improvement suggested by the experts concerns specifically the graph from Fig. 17. In this graph, we observe that the *diabetes mellitus* concept is dealt as a specific concept of the generic *mellitus* concept. This is not true, as *diabetes mellitus* represents a concept (a disease). It is not supposed to be broken down as the result obtained by our technique. This happened because the dependency parser from Stanford CoreNLP toolkit considered both *diabetes* and *mellitus* as separate nouns. Also, *diabetes* is a compound linked to *mellitus*. In practice, this could be mitigated by incorporating a biomedical named entity recognizer (NER) to the technique. If such NER identifies *diabetes mellitus* as a whole entity, there would be no break it down. Another option would be using a dependency parser trained in biomedical texts, that would prevent such an issue.

Most models used in NLP tools and techniques are trained in a very large, but finite set of texts. Due to new findings and investigation works, the biomedical domain evolves quickly and new entity names are introduced to the vocabulary. For this reason, the trained models require timely updates to catch up to the state of the art. Therefore, most NLP tools and techniques may always fail in some aspect, being it either recognizing named entities, identifying parts of speech, or generating parse trees. This enforces the use of a semi-automatic method, where such limitations on tools and techniques may be overcame by manual interaction when required.

Furthermore, there may be minor human errors in the texts (e.g., wrong punctuation, ambiguous sentences, *etc*.) that may also interfere with the output of NLP tools and techniques. Such erroneous outputs might interfere with the generation of RDF triples, and in consequence, generate erroneous KGs. For this reason, a semi-automatic approach is valuable, as an expert might be able to review the method’s overall and intermediary outputs, and interfere with the process, so that we may have the most appropriate outputs.

### Experiment II: ontology links

In this evaluation, we compared the ontology links obtained from the same unstructured input text, between KGs generated using the ontology linking method from our previous work [14], against the updated linking method proposed and implemented in this work.

The previous method consists on retrieving the annotations generated from the National Center for Biomedical Ontology (NCBO) bioportal, by means of their REST APIs, where we passed in as parameters, the actual text to be annotated, and a target ontology, from which the links should be obtained.

The new method, on the other hand, consists in using a trained model able to recognize biomedical named entities, and a NLP Part of Speech (PoS) tagger to recognize verbs. The model is able to provide UMLS CUIs, for the given recognized entities and verbs. Then, by using NCBO bioportal’s SPARQL endpoint, we query the corresponding matches for such concept IDs in a target ontology.

The comparison of both methods used the same conclusions sub-sections of the abstracts used in Experiment I, labeled from *A00* to *A05*. The target ontology selected is NCIT, the same ontology from the examples in Section . NCIT is the most suitable ontology, as it is suggested by NCBO recommender service [59] for all the abstracts texts. The trained model, which provides NER and UMLS CUIs, was obtained from the ScispaCy project [57].

We first compared the amount of links obtained using both methods, in terms of number of links generated. Table 5 summarizes the results. We observe that for all abstracts, the new method shows better results in obtaining links.

**Table 5:** Comparison between ontology linking methods

	A00	A01	A02	A03	A04	A05
Previous method	4	3	6	5	4	6
New method	6	8	11	10	9	9

Amount of links found for the previous method (retrieved from the NCBO annotator), and the new method (obtained through ScispaCy trained model and UMLS SPARQL queries)

Considering the difference between the links obtained in both methods, we have links that were added (i.e., they did not exist in the previous method), links that were maintained (i.e., they exist in both the previous and the new method), and links that were removed (they exist in the previous method, but no longer exist in the new method). Table 6 presents such cases.

**Table 6:** Analysis of changes in links provided by the through ScispaCy trained model and UMLS SPARQL queries

	A00	A01	A02	A03	A04	A05
Added	4	6	6	8	5	7
Maintained	2	2	5	2	4	2
Removed	2	1	1	3	0	4

Amount of links added, maintained, and removed, when comparing the previous method and the proposed method

The amount of links found for the new method in Table 5 considers the links obtained in the target ontology. As already explained, before obtaining the final link, the method first identifies the term (a named entity or a verb), then obtains a UMLS CUI, and finally, obtains the target ontology link through a SPARQL query. Therefore, there may be some cases where: (1) the term is identified and there is no UMLS match; (2) the term is identified, a UMLS CUI is found, but there is no match in the target ontology; and (3) the most successful case, where the term is identified, a UMLS CUI is found, and a match in the target ontology is also found. Such cases are presented in Table 7.

**Table 7:** Steps in generating links in the new method

	A00	A01	A02	A03	A04	A05
Terms identified	17	17	21	21	18	26
UMLS CUIs found	11	10	14	14	11	18
Target Ontology links	6	8	11	10	9	9

Number of terms identified (named entities and verbs), UMLS CUIs that are found for these terms, and links to a target ontology obtained for these UMLS CUIs

The numbers presented in Table 7 indicate that if we choose a different target ontology, we may find different results, as the final link is obtained through an existing mapping between the target ontology and UMLS. If we still consider the same target ontology, one possible direction to improve the results would be combining the results for both methods, i.e., adding the target ontology links obtained from the previous method (NCBO annotator) that are not found through the UMLS method to the output. The results may also be possibly further enhanced if we specifically search for the identified terms, that neither have a corresponding UMLS CUI, nor an annotated result in the target ontology.

We updated our KGen tool to incorporate and evaluate this combined approach to further enhance the results. Table 8 shows the updated results when performing this approach. We found a positive outcome, as the number of links increased.

**Table 8:** Further comparison between ontology linking methods

	A00	A01	A02	A03	A04	A05
Previous method	4	3	6	5	4	6
New method	6	8	11	10	9	9
Combination	8	11	12	12	11	13

Amount of links found for the previous method (using the NCBO annotator), the new method (obtained through ScispaCy trained model and UMLS SPARQL queries), and a combination of both methods

## Discussion

This investigation defined, developed, and evaluated KGen, a semi-automatic method to generate KGs from natural language texts from biomedical scientific literature using NLP techniques. The method advances the state of the art in extracting not only the main relations from sentences and representing them as RDF triples, but also secondary relations, using the output of a dependency parser. We introduced a technique to link entities and relations from the KG to concepts and properties in biomedical ontologies. Our technique explored a trained model that recognizes biomedical named entities that are mapped to concepts and properties in the UMLS semantic network. Such concepts and properties are, in turn, mapped to the ones from a targeted ontology by means of SPARQL queries. The benefit of this mapping is to enable the direct comparison of concepts on different KGs, bringing them to a common basis of comparison.

We conducted experiments to evaluate the quality of the proposed ontology linking method, and the quality of the RDF triples generated by KGen. The triples evaluation involved two physicians, who manually generated triples from six abstracts from papers related to the Alzheimer’s disease. From the same six abstracts, we ran our tool that implements KGen method. We discovered that KGen is able to extract more triples than the physicians, due to the main and secondary relations that it identifies and extracts from the text sentences. The comparison was performed using the Jaccard similarity coefficient. It can be seen as a proxy to an accuracy assessment, which cannot be directly performed due to the lack of a gold standard in the current stage of this research. The Jaccard coefficient denotes how similar are the sets of triples extracted by KGen, in comparison to the sets manually extracted by the physicians.

Although the lack of a gold standard and a direct accuracy assessment is a limitation of our work, as direct measurements could lead to more robust conclusions, the use of the Jaccard similarity coefficient shows promising results regarding the utilization of our approach.

The physicians did not directly verify the triples generated by KGen nor performed any comparison against their manually-generated triples. All the analyses were conducted by the authors. Our analysis identified that physicians were able to extract non-trivial triples from texts, which involves logical thinking and previous knowledge in the area. The semi-automatic nature of KGen approach enables to combine both the automatically extracted set of triples with the manually extraction. The benefits in this approach refers to the ability in generating an enriching KG that combines the explicit knowledge represented in the text with experts’ implicit knowledge suited to derive from the same text.

In addition, the fact that the involved physicians in this study are surgeons, and not AD specialists, also represents a limitation of our work. We believe that the involvement of AD experts could, of course, enrich and strength our conclusions. However, the ultimate objective of our proposed method is to generate a knowledge representation of texts, that is expected to be useful not only for experts in a particular domain, but also for researchers, physicians, and even students with background knowledge in related areas. The involvement of the surgeon physicians in this study is therefore aligned to our objective. As the subjects considered in our study have a broader knowledge about the target domain, we believe that their assessments are still valid in our attempt to confirm that our approach leads to relevant knowledge representations of texts in the biomedical domain.

The ontology linking evaluation involved a direct comparison between the proposed ontology linking approach, and the annotations generated by the NCBO webportal. Our refined proposed linking approach showed better results in terms of the amount of links found. We found a small overlap in terms of links detected by both methods. In a few cases, though, there are links found by the annotator, which are not detected by the proposed method. We noticed that it is possible to combine links generated by both methods to produce an enhanced result, which further increases the overall amount of links obtained for a target ontology.

The language employed on scientific papers, especially those in the degenerative diseases domain, poses a great difficulty for techniques and tools involved in the method. Furthermore, texts may also present problematic constructions, in terms of punctuation, spelling, or even ambiguous sentences. For this reason, a fully automated method is still an open research challenge. Although our method is able to run to completion without human intervention, the method allows a domain specialist to review and manually change the intermediate artifacts, i.e., the preprocessed text, triples, ontology links, and the RDF representation of the KG. In the KGen tool, such intermediary artifacts are represented by text files. When they are manually changed, the tool is able to reconsider those intermediary files and update the resulting graphs.

In the current implementation of KGen tool, we used python programming language, and a variety of NLP tools and models combined for tasks. For instance, named entity recognition explored ScispaCy’s models that are targeted for biomedical texts. Although such models and tools showed satisfactory results in the processed texts considered in the current evaluation, it is important to mention that our solution provides flexibility for changing the models used, or even the tools employed. That will be explored in future evaluations.

An aspect that could be further investigated is an automatic approach to generate RDF triples more similar to the ones generated by domain experts. Obtaining relations that are rather implicit in the text sentences could rely on logical inferences. Such inferences could be derived from the main and secondary automated triples, by using, for instance, machine learning approaches. Triples that require a previous knowledge to be generated, on the other hand, could be harder to derive. A possible investigation would be by using other KGs generated from texts in the same area, as well as ontologies in the domain. SPARQL queries could be employed for finding related concepts or properties, and, thus, further enrich the primary triple set. Another possibility lies on using inference reasoning in the primary triple set to derive a secondary triple set.

Another interesting venue of research lies on the intermediary UMLS mappings for the entities and relations represented in the graph. SPARQL queries using the UMLS CUIs could be of great assistance when comparing KGs that linked to different ontologies. The UMLS CUIs could be used as a common ground in such comparison, or even be used to generate additional mappings to more than one ontology, enabling a fair comparison between such different KGs.

The analysis performed by comparing KGen’s triples to the triples manually extracted by experts brings another possible venue for future investigations. We plan inviting other domain experts in the biomedical domain to manually extract triples from similar texts and help us on generating a comparison baseline for methods that aim on generating ontology-linked KGs.

## Conclusion

The generation of ontology-linked KGs from unstructured texts is still an open research challenge. When considering texts from the biomedical scientific literature, and computational ontologies in the biomedical domain, additional challenges are imposed. Ontology-linked KGs can benefit further integration and understanding of research findings by turning possible queries over structured data. In this article, we proposed a method to semi-automatically generate ontology-linked KGs from texts in the biomedical scientific literature. The method extracts main and secondary relations from text sentences, representing them in form of triples. Biomedical named entities and relations are identified and linked to concepts and relations from ontologies in the biomedical domain. We conducted experiments involving domain experts to evaluate the quality of the generated RDF triples. We carried out direct comparison between ontology linking methods to ensure that the mappings to target ontologies are properly achieved in the KG generation. The results showed that the method successfully achieves its objectives in identifying and representing relations obtained from text sentences. Ontology-linked KGs were properly obtained containing ontology links, thus, affirmatively answering our research question. Future work involves the study and comparison of temporal KGs, i.e., ontology-linked KGs generated from scientific texts in different time stamps.

## Data Availability

All source code and data developed/used in this study are available at: https://github.com/rossanez/kgen

## Abbreviations

KG: Knowledge Graph;
RDF: Resource Description Framework;
URI: Universal Resource Identifier;
NL: Natural Language;
NLP: Natural Language Processing
UMLS: Unified Medical Language System;
CUI: Concept Unique Identifier;
NCBO: National Center for Biomedical Ontology;
NCIT: National Cancer Institute Thesaurus;
AD: Alzheimer’s Disease;
ADO: Alzheimer’s Disease Ontology;
PoS: Part of Speech;
NER: Named Entity Recognition;
SRL: Semantic Role Labeling;
CNN: Convolutional Neural Network;
RNN: Recurrent Neural Network.
